# The Multifaceted Roles of Eukaryotic Elongation Factor 1‐Alpha‐2 in Neurodevelopmental and Neurodegenerative Disorders

**DOI:** 10.1155/tswj/4373377

**Published:** 2026-05-04

**Authors:** Kawinthra Khwanraj, Athinan Prommahom, Permphan Dharmasaroja

**Affiliations:** ^1^ Chakri Naruebodindra Medical Institute, Faculty of Medicine Ramathibodi Hospital, Mahidol University, Samut Prakan, Thailand, mahidol.ac.th

**Keywords:** autism spectrum disorder, eEF1A2, epilepsy, neurodegeneration, neurodevelopment, Parkinson′s disease

## Abstract

Eukaryotic elongation factor 1‐alpha‐2 (eEF1A2) is a neuron‐ and muscle‐specific translation elongation factor isoform that supports high‐demand proteostasis in terminally differentiated cells. Beyond its canonical role in translation elongation, eEF1A2 participates in noncanonical processes linked to actin cytoskeleton regulation, compartmentalized/local translation, and stress–response signaling. A central challenge in the field is that the strength and type of evidence implicating eEF1A2 differ substantially across disease classes. In neurodevelopmental disorders, heterozygous de novo pathogenic variants in EEF1A2 provide strong causal human genetic evidence for developmental and epileptic encephalopathies and related phenotypes, supported by functional studies showing reduced de novo protein synthesis/elongation and altered actin bundling in common patient‐associated variants. In contrast, in neurodegenerative paradigms (e.g., toxin‐based Parkinson′s disease models and ischemia‐reperfusion injury), eEF1A2 is primarily implicated as a contributory node within oxidative stress, autophagy/mitophagy, and inflammatory signaling pathways, largely based on cellular and animal model evidence rather than Mendelian causality. This review adopts an evidence‐based framework and organizes findings around a unified mechanistic model connecting canonical elongation and noncanonical cytoskeleton/stress/autophagy functions to neuronal phenotypes. Collectively, current evidence supports a causal role for eEF1A2 in neurodevelopmental disorders but only a contributory role in neurodegenerative conditions.

## 1. Introduction

The development of neuronal cells, synaptic plasticity, and neural circuit maintenance depend on the coordinated regulation of protein synthesis, cytoskeletal dynamics, and stress resistance. Translation elongation factors, such as eEF1A isoforms, are traditionally classified as “housekeeping” genes, but specialization of isoforms in differentiated tissues indicates the involvement of the translation elongation machinery in functional integration with other signal transduction and cytoskeletal systems [1]. Eukaryotic translational elongation factor 1‐alpha‐2 (eEF1A2) is a protein specifically expressed in neuronal and muscle tissues and is increasingly recognized as a translation/cytoskeleton/stress integrator rather than a ribosome‐associated protein.

The human *EEF1A2* gene is located on the Chromosome 20q13.3 and has been well studied through genomic sequencing techniques, and the structural organization of the gene has been understood [2]. The human *EEF1A2* gene is approximately 10 kb in size and consists of eight exons. The boundaries of the exons and introns show high levels of conservation. Primer extension analysis of the human *EEF1A2* gene has revealed the transcription start site of the gene to be 166 base pairs upstream of the AUG start codon [3]. This provides a foundation for understanding its transcriptional regulation. Analysis of the promoter region of the *EEF1A2* gene has identified several critical *cis*‐regulatory elements (CREs) that are essential for the regulation of the gene. These include the presence of E‐box elements, early growth response (EGR) protein‐binding sites, a GATA motif, and a myocyte enhancer factor‐2 (MEF2) DNA‐binding site [3]. These elements indicate that the transcription of the *EEF1A2* gene is regulated by transcription factors that are involved in cell differentiation and tissue‐specific functions.

To ensure conceptual clarity and consistency of concepts across disease contexts, this review has employed an evidence‐based approach that differentiates between the causal and contributory effects of eEF1A2. In the context of neurodevelopmental disorders, the discussion has been underpinned by robust causal evidence from replicated human genetic studies, particularly those of recurrent de novo pathogenic variants that are functionally validated. In contrast, in neurodegenerative disorders, eEF1A2 is discussed primarily as a contributory factor within broader pathological pathways, where current evidence is largely derived from mechanistic evidence, obtained from cellular and animal models (e.g., oxidative stress, autophagy, and receptor coupling), rather than Mendelian causality or direct human genetic causation.

## 2. Characterization of the Human eEF1A2 Gene and Protein

The characterization of the *EEF1A2* gene has been facilitated by a variety of different approaches, including genomic sequencing, promoter activity assays, proteomics, and functional validation studies. These studies collectively highlight the unique regulation of the *EEF1A2* gene and the functional significance of the eEF1A2 protein.

### 2.1. Genomic Sequencing

By utilizing overlapping clones of genomic DNA from human genomic libraries, the location of the *EEF1A2* gene was mapped and sequenced. Such studies revealed that the gene is approximately 10 kb long and contains eight exons [3]. In addition, the intron–exon boundaries are highly conserved. However, there are relatively long intronic sequences within the gene, which may indicate a complex regulation of transcription. Furthermore, primer extension analysis revealed that the transcriptional start site of the *EEF1A2* gene is located 166 base pairs upstream from the AUG translation initiation codon. These studies collectively indicate the overall conservation of the *EEF1A2* gene across different species.

### 2.2. Promoter Activity Assays

The analysis of the *EEF1A2* promoter region also revealed the presence of several CREs, which play an essential role in the transcriptional activity of the *EEF1A2* promoter region. These CREs include 12 E‐box elements, 3 EGR transcription factors, 1 GATA transcription factor, and 1 MEF2 transcription factor [3]. These transcription factors play a crucial role in various cellular processes. Analysis of the *EEF1A2* promoter region by the progressive deletion of constructs revealed the presence of the minimal promoter region of the gene from −16 to +92 base pairs of the transcription start site of the promoter region. This is crucial for the transcriptional activity of the *EEF1A2* promoter region. This has opened the doors for further research in the transcriptional regulation of the *EEF1A2* gene in a tissue‐specific manner.

### 2.3. Proteomics, Structural Analysis, and Posttranslational Modifications (PTMs) of eEF1A2

Comprehensive proteomic and structural analyses have identified important PTMs and structural elements that play a critical role in governing the specific functions of eEF1A2. Using high‐throughput mass spectrometry techniques, a number of PTMs have been reported for eEF1A2, such as phosphorylation, methylation, acetylation, ubiquitination, and S‐nitrosylation; many of these PTMs have also been found to be localized to specific regions of the eEF1A2 protein surface, often termed the “variable face” of the protein [4]. These PTMs, especially those occurring on lysine and arginine residues, are related to charge redistributions that could affect eEF1A2 function.

Moreover, structural modeling of eEF1A based on homology has also revealed clusters of PTMs on the surface of the protein where sequence divergence has occurred [5]. These clusters are related to areas rich in PTMs and are associated with the interaction of the protein with cellular components such as actin and signaling molecules. Therefore, this has highlighted the potential role of PTMs in the regulation of the canonical and noncanonical functions of eEF1A2. In this regard, some of the PTMs, such as phosphorylation sites (e.g., S358), have been experimentally confirmed [4].

In addition, structural studies utilizing molecular dynamics simulations have shown that eEF1A2 exists in reversible “open” and “closed” conformations, which could play a role in the regulation of eEF1A2 binding to translation and cytoskeleton components. eEF1A2 has a lower binding affinity for calmodulin than its related eEF1A isoform because of reduced flexibility in the region of eEF1A2 that binds to calmodulin; this probably makes eEF1A2 more stable and specific for neuronal functions [6]. These results demonstrate the importance of the structural properties of eEF1A2 in its localization in high translation–demanding areas, such as the synapse.

In conclusion, the results demonstrate the importance of the structural properties and PTMs of eEF1A2 in the functional versatility of the protein, especially in the context of the regulation of the cytoskeleton and cell signaling.

## 3. eEF1A2 Biology in the Nervous System

### 3.1. Expression Timing and the “Wasted” Mouse Model

**Figure 1:**
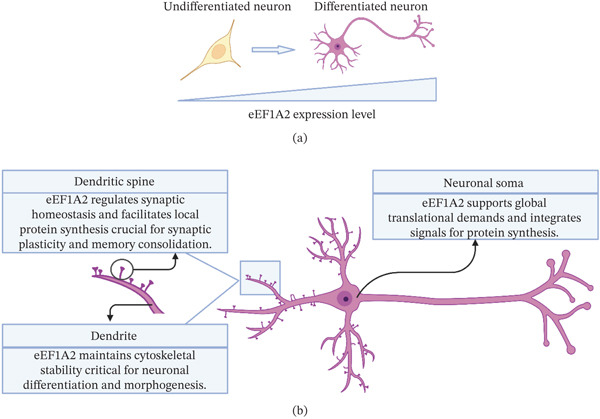
(a) The dynamic expression of eEF1A2 during neuronal differentiation and (b) its functional roles in the mature neuron. As neurons mature, eEF1A2 expression increases, enabling its localization to specific subcellular compartments (dendritic spine, dendrite, and soma) to support vital processes such as synaptic plasticity, cytoskeletal integrity, and protein synthesis.

eEF1A2 has a developmentally regulated and tissue‐specific expression profile, becoming enriched in the brain, heart, and skeletal muscle postnatally. This is supported by the fact that its expression is upregulated during the postnatal development of the central nervous system (CNS), as evidenced by immunohistochemical staining and gene‐edited mice [7, 8]. This emphasizes that it is required to support the functions of terminally differentiated neurons (Figure 1a). One of the major genetic models that helps understand the consequences of the expression pattern during development is the “wasted mouse” or *wst/wst* mutation, which is a spontaneous autosomal recessive mutation involving the loss of eEF1A2 expression. The mice follow a normal pattern of development, but by the 21st day, there is a rapid onset of neuromuscular pathology and death by the 28th day of age [9]. The investigation in wasted mice reveals that the lack of eEF1A2 leads to a “dying‐back” neuropathy in the neuromuscular junction, where the neuropathy begins in the synapse, followed by axonal degeneration [10]. This neuropathy has a distinct morphology compared with the classical Wallerian degeneration. These data indicate that the postnatal expression of eEF1A2 is critical for the maintenance of neuronal and neuromuscular integrity.

### 3.2. Subcellular Distribution and Localization

The localization of the eEF1A2 is dynamic and context‐dependent and is influenced by development and neuronal activity (Figure 1b). Its localization is commonly found in high levels in the soma of the neuron and is proposed to be correlated with the proteostasis of long‐lived differentiated cells. However, the localization of the eEF1A2 is not exclusive to the neuronal soma. Biochemical and localization studies have identified eEF1A2 as a binding partner of the M4 muscarinic receptor, with colocalization observed in both soma and neuropil in the rat striatum [11]. In addition, transcript‐level analyses suggest that subcellular localization may be influenced by methodological factors such as antibody specificity. This supports the use of RNA‐based approaches to better resolve its distribution [12].

Using single‐molecule fluorescence in situ hybridization and RNA localization analyses, these studies show that eEF1A2 is expressed at later stages of neuronal development and in mature neurons, and its localization is region‐ and compartment‐specific [12]. These observations suggest the role of eEF1A2 in the maintenance of neuronal structures and functions beyond the neuronal cell body. The role of eEF1A2 is also related to the regulation of translation and synaptic plasticity, especially in dendritic compartments of high translational need. Experimental studies have demonstrated that eEF1A2 participates in the regulation of protein synthesis and actin dynamics in dendritic spine remodeling through the phosphorylation of isoform‐specific sites of eEF1A2 [13].

These findings indicate that eEF1A2 has a dual function in the regulation of global and local protein synthesis, which is important in supporting synaptic function and stability.

## 4. Mechanistic Framework: Linking eEF1A2 Molecular Functions to Neuronal Phenotypes

This section consolidates mechanistic evidence into a unified framework and explicitly links molecular effects to cellular phenotypes and, where supported by evidence, to circuit‐ and organism‐level outcomes. The role of eEF1A2 is better described as an integrator that links canonical translation elongation processes, specifically the delivery of aminoacyl tRNA to the A site of the ribosome, to noncanonical functions that are associated with neuronal morphogenesis and synaptic adaptation processes. These include the binding and bundling of actin, compartmentalized or local translation in dendrites and spines, the modulation of cellular stress responses, and autophagy and mitophagy processes. [8]. PTMs, such as phosphorylation at isoform‐specific sites, have been shown to regulate the function of eEF1A2 by switching between translation and cytoskeleton remodeling processes, providing a molecular basis that links synaptic stimulation and structural plasticity. [13]. In neurodevelopmental diseases, the function of the de novo mutation in the *EEF1A2* gene has been associated with decreased levels of de novo protein synthesis and altered actin regulation in neurons, providing a putative disease pathway from molecular dysfunction to synaptic and circuit dysfunction. [14].

### 4.1. Canonical Elongation and Protein Synthesis Capacity

eEF1A2 transports aminoacyl‐tRNA to ribosomes during translation elongation. An elongation defect in neurons ranges from decreased global protein synthesis to decreased translation capacity in compartments, which may impact synapse formation and plasticity in response to neuronal activity. The functional analysis of variants commonly found in patients (G70S, E122K, and D252H) revealed decreased de novo protein synthesis and elongation rate in cell culture systems (e.g., HEK293), offering direct mechanistic insights into translation dysfunction [14].

### 4.2. Noncanonical Roles of eEF1A2 in Cytoskeletal Regulation and Neuronal Morphology

Apart from its conventional role in translation, eEF1A2 also performs crucial noncanonical roles, including cytoskeletal regulation and neuronal structure. Indeed, experimental studies have shown that disease‐related mutants (G70S, E122K, and D252H) not only negatively regulate protein translation but also cytoskeletal bundling and neuronal morphology, thereby supporting a model for a functional connection between protein synthesis and cytoskeletal dynamics, which are crucial for synaptic development and neuronal excitability [14]. The molecular basis for mutant proteins of eEF1A2 includes changes in their tRNA binding and actin bundling, which result in abnormal cytoskeletal structure and development.

Further evidence for these noncanonical roles comes from in vivo experiments. Mouse models created by using CRISPR/Cas9 technology to introduce biallelic *Eef1a2* mutations show severe neurodevelopmental phenotypes, such as seizures, degeneration of motor neurons, and death shortly after birth, accompanied by a loss of neurons in brain regions such as the hippocampus [15]. Notably, some mutations, such as G70S, show a lack of rescue of neurodegeneration, suggesting impaired function, whereas others, such as D252H, show complex mechanisms involving loss‐ and gain‐of‐function effects, as suggested by more severe phenotypes than those seen for null mutations and disrupted interactions with proteins, respectively [16]. In addition, in vivo studies demonstrate that targeted overexpression of eEF1A2 significantly enhances corticospinal axon sprouting after CNS injury, in association with mTOR signaling, protein synthesis, and regulation of the actin cytoskeleton [17].

In terms of genomic variation, structural changes in *EEF1A2*, including duplications and deletions in the 20q13.33 region and ring Chromosome 20, have been linked to developmental delay and seizures and may affect biological processes including microtubule dynamics, nucleosome assembly, DNA repair, and synaptic transmission, further supporting the central role of eEF1A2 in neurodevelopmental disorders [18].

### 4.3. PTMs as Functional Switches

It has been found that the PTMs play an important role in the regulation of the function of eEF1A2. Various proteomic studies have confirmed that the PTMs, like phosphorylation, methylation, and acetylation, in different regions of the surface of eEF1A2, are responsible for the interaction with other biomolecules [4]. For example, the phosphorylation of certain sites, like S358, is responsible for the interaction with the cytoskeleton, especially actin, and the signaling pathways. This interaction is very important for the stability of neurons and synaptic function.

In the context of neuronal cells, PTMs have been described to play the role of functional switches in regulating the functions of eEF1A2. For hippocampal neurons, the phosphorylation of isoform‐specific sites has been observed to influence the dissociation of eEF1A2 from its regulatory partners and to regulate the functional balance of the protein in the context of translation and actin dynamics in remodeling dendritic spines [13]. Other PTMs, including lysine methylation and acetylation, have also been described to regulate the structural stability and interaction networks of the protein, thereby ensuring the integration of protein translation and actin dynamics [4].

Based on this evidence, the direct link between PTMs and human diseases at different stages and progression is limited, and it is best to consider the mechanisms of PTMs as possible mechanisms of regulation rather than the causation of diseases.

### 4.4. Receptor‐Coupled Regulation of eEF1A2

The role of eEF1A2 in receptor‐associated signaling is mediated via interaction with muscarinic acetylcholine receptors (mAChRs), with special emphasis on the M4 subtype of mAChRs. This interaction suggests a link between neuronal signaling and translation regulation. The biochemical and localization studies have confirmed the direct interaction of eEF1A2 and the M4 mAChR by colocalization in the soma and neuropil regions of the brain, thereby establishing a basis for compartmentalized regulation [11, 19]. The M4 mAChR has been established as a guanine nucleotide exchange factor (GEF) for eEF1A2 by direct binding to the receptor′s intracellular loop region, termed M4i3, which modulates its activity beyond canonical cytosolic translation [11].

This interaction implies a possible role for eEF1A2 in the regulation of actin bundling and microtubule‐associated processes, which are essential for synaptic activity and plasticity. In light of the fundamental role of mAChR‐mediated signaling in cognition and motor functions, as well as in neurological disorders such as Parkinson′s disease (PD), Alzheimer′s disease (AD), and schizophrenia [20–22], a possible role for eEF1A2 as a downstream target of this pathway in cellular processes is implied.

### 4.5. Stress Response, Oxidative Stress Resilience, and Apoptosis

The role of eEF1A2 in various stress responses, including oxidative stress resistance and apoptosis, has been investigated in various experimental studies. The interaction of eEF1A2 with the antioxidant protein Peroxiredoxin 1 (PRDX1) has been observed to have a critical role in the development of resistance to peroxide‐induced cell death via mechanisms including reduced caspase activation and increased activation of prosurvival pathways such as Akt activation [23–27]. In toxin‐induced models using 6‐hydroxydopamine and MPP^+^ treatment, eEF1A2 expression was found to be dynamically regulated in response to oxidative stress, and its interaction with antioxidant proteins in neurons was found to play an important role in conferring oxidative stress resistance [23, 26, 27]. Moreover, human genetic studies have provided insights into how *EEF1A2* variants may confer susceptibility to disorders through their effects on protein synthesis and integrated stress response pathways, providing a potential link to early neurodevelopmental abnormalities and progressive or stress‐induced susceptibility in neurons [28].

### 4.6. Autophagy/Mitophagy Regulation in Neuronal Stress Models

eEF1A2 plays a critical role in regulating autophagy, mitophagy, and neuronal survival under stress conditions. In toxin‐based dopaminergic models, particularly MPP^+^‐treated differentiated SH‐SY5Y cells, knockdown of *EEF1A2* impairs autophagic flux, as evidenced by reductions in LC3 puncta, LC3‐II/LC3‐I ratio, and phosphorylated Beclin‐1 levels [29]. These changes are accompanied by increased mitochondrial damage, defective mitophagy, and *α*‐synuclein accumulation, indicating disrupted protein degradation pathways and compromised cellular homeostasis. Further mechanistic studies demonstrate that eEF1A2 deficiency suppresses key survival signaling pathways, including Akt1 and mTORC1, while increasing proapoptotic markers such as p53 and caspase‐3, thereby exacerbating neuronal cell death under oxidative stress conditions [30]. Conversely, feedback regulation within the Akt/mTOR pathway may influence eEF1A2 expression, suggesting a dynamic role in stress adaptation.

eEF1A2 is involved in the modulation of autophagy, mitophagy, and survival pathways in neurons subjected to stress. In toxin‐induced models of dopaminergic neurons, especially in MPP^+^‐treated differentiated SH‐SY5Y cells, the knockdown of *EEF1A2* has been associated with the impairment of autophagy, which is characterized by reduced expression of LC3 puncta, LC3‐II/I, and phospho‐Beclin1 [29]. This is associated with mitochondrial damage, defective mitophagy, and the accumulation of *α*‐synuclein, indicating that protein degradation is impaired and cellular homeostasis is compromised. Further research has indicated that the deficiency of eEF1A2 is associated with the reduction of survival signaling molecules such as Akt1 and mTORC1, and the upregulation of apoptosis mechanisms such as p53 and caspase‐3 [30]. In contrast, the expression of eEF1A2 is also influenced by the regulatory feedback within the Akt/mTOR pathway, suggesting a dynamic role in stress adaptation.

Recent studies have also found that eEF1A2 is an important regulator of the immunoproteasome under mitochondrial stress. Mitochondrial dysfunction leads to the accumulation of nonimported mitochondrial precursor proteins in the cytoplasm, which pose a significant threat to cellular proteostasis. To reduce this proteotoxic stress, human cells lacking mitochondrial Complex I increase the levels of eEF1A2, leading to increased production of the immunoproteasome‐specific subunit PSMB9 and the small heat shock protein HSPB1 [31]. The mechanistic role of eEF1A2 is its regulation of the transcription and translation of PSMB9. This results in specialized proteasome complexes, which are targeted to damaged mitochondria for protein degradation. Depletion of eEF1A2 results in a dramatic increase in protein aggregation and a negative effect on neuronal function.

These results are consistent with a protective role for eEF1A2 in the context of autophagy‐ and mitophagy‐mediated stress mitigation in models of neuronal stress. Nevertheless, this evidence is derived from the level of pathways and does not prove genetic causality.

### 4.7. Translatome Profiling and Targets of eEF1A2

Existing functional studies demonstrate altered global protein synthesis in patient‐associated variants, including evidence from puromycin‐based assays (e.g., SUnSET/SuNRISE) and coupling to synaptic translation processes [14]. However, the field currently lacks a well‐defined, neuron type–specific “eEF1A2‐dependent translatome signature,” and there is no widely accepted catalog of isoform‐specific target mRNAs analogous to those described for canonical RNA‐binding proteins. Any apparent target specificity is therefore more plausibly explained by mechanisms such as compartmentalized translation, interaction with regulatory partners, or selective engagement of stress–response programs.

To fill the existing gaps, different existing experimental approaches can be adopted to investigate eEF1A2 variants. For example, it is possible to carry out an in‐depth analysis of translation at the nucleotide level using the method of ribosome profiling [32]. Alternatively, different translatome capture approaches, such as TRAP and RiboTag, have been developed to capture ribosome‐bound mRNAs from specific neurons using different cell type–specific approaches in vivo [33]. In addition, puromycin‐based approaches, including SUnSET, have been developed to quantify changes in translation in specific populations of cells [34]. Together, these approaches define clear experimental strategies for characterizing eEF1A2‐dependent translation processes while avoiding unsupported claims of discrete molecular targets.

**Figure 2:**
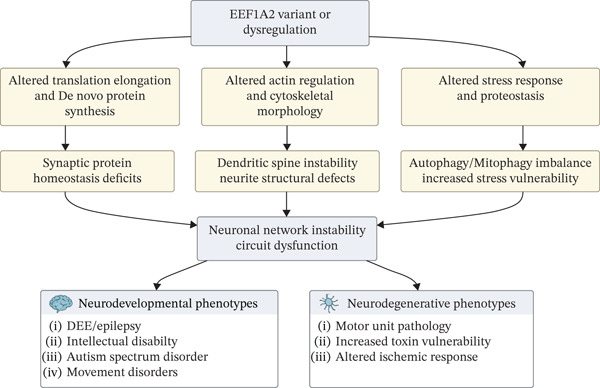
Mechanistic diagram of eEF1A2 dysregulation in neurodevelopmental and neurodegenerative disorders. Dysregulation of eEF1A2 disrupts translation, cytoskeletal regulation, and proteostasis, leading to synaptic deficits, spine instability, and autophagy imbalance. These changes converge on neuronal network dysfunction, resulting in neurodevelopmental and neurodegenerative phenotypes.

From a mechanistic standpoint, *EEF1A2* variants or dysregulation lead to disruption of fundamental processes within neurons, such as translation elongation, actin cytoskeletal dynamics, and stress responses, leading to defective de novo protein synthesis, neuritic instability, and proteostasis imbalance (Figure 2). These cellular defects converge to synaptic protein homeostasis disruption, autophagy/mitophagy dysfunction, and impaired stress responses, leading to instability of the neural network. Collectively, these disturbances could manifest as abnormal neurodevelopmental and neurodegenerative disorders.

## 5. Neurodevelopmental Disorders Causally Linked to eEF1A2

### 5.1. Evidence Summary

Independent studies have identified heterozygous de novo variants in *EEF1A2* in patients who display developmental delay, intellectual disability (ID), autism spectrum features, epileptic encephalopathy, and movement disorders [28, 35]. This has confirmed the genotype‐spectrum correlation for a dominant neurodevelopmental syndrome. Studies of recurrent variants, such as G70S, E122K, and D252H, in *EEF1A2*, which reduce de novo protein synthesis and elongation, as well as affect actin bundling and neuronal morphology, highlight the links between disrupted translation and cytoskeletal regulation [14].

Table 1 integrates these data by mapping representative variants to their inheritance, clinical phenotypes, and both canonical (translation‐related) and noncanonical (cellular or cytoskeletal) effects. Across variants, a common theme emerges of hypomorphic translation defects coexisting with variant‐specific cellular perturbations, including altered tRNA binding, cytoskeletal dysregulation, and neuronal toxicity. These observations suggest that eEF1A2‐associated disorders cannot be explained by a single mechanism. Because neuronal development and synaptic plasticity require tightly regulated translation–actin coordination, both reduced elongation capacity and inappropriate cytoskeletal coupling can converge on shared network‐level outcomes (seizure susceptibility and developmental delay), consistent with a homeostasis disruption.

**Table 1:** Variant–function mapping for representative *EEF1A2* variants.

Variant (protein)	Inheritance	Reported phenotype	Canonical effect	Noncanonical effect	Mechanistic interpretation	Source
p.Gly70Ser (G70S)	De novo heterozygous	DEE/epilepsy; ID; ASD features	↓ De novo protein synthesis; ↓ elongation rate	↑ tRNA binding; ↓ actin bundling; altered neuronal morphology	Hypomorphic elongation with dysregulated/toxic GoF properties possible	[14, 35]
p.Glu122Lys (E122K)	De novo heterozygous (recurrent)	Early‐onset epilepsy/DEE; severe ID; EEG features reported	↓ De novo protein synthesis; ↓ elongation rate	↓ Actin bundling; altered neuronal morphology	Hypomorphic elongation + altered cytoskeletal coupling; variant‐specific network effects plausible	[14, 36]
p.Asp252His (D252H)	De novo heterozygous	ASD/ID ± epilepsy	↓ De novo protein synthesis; ↓ elongation rate	Altered neuronal morphology; toxicity in neuronal assays	Mixed mechanism plausible in dominant missense disease; toxic GoF	[14]
p.Pro333Leu (P333L) (biallelic)	De novo heterozygous	Severe multisystem; early death	Likely severe loss	Developmental lethality models	Recessive LoF	[28]
Multiple de novo missense variants	De novo heterozygous	Epileptic − dyskinetic encephalopathy ± degenerative course	Protein‐damaging in complementation assays; translation/ISR disruption proposed	Integrated stress response disruption proposed	Haploinsufficiency proposed at cohort level; variant‐specific effects likely	[28]

Abbreviations: ASD, autism spectrum disorder; DEE, developmental and epileptic encephalopathies; EEG, electroencephalography; ID, intellectual disability; IRS, integrated stress response; GoF, gain of function; LoF, loss of function.

### 5.2. Epilepsy and Developmental and Epileptic Encephalopathy (DEE)

eEF1A2‐associated epilepsy ranges from severe DEEs to milder phenotypes, indicating marked variable expressivity [35, 36]. Human genetic studies using whole‐exome sequencing and cohort analyses have consistently demonstrated de novo *EEF1A2* variants, such as G70S, E122K, and D252H, in patients who presented with seizures, severe developmental delay, ID, hypotonia, and autistic features [28, 37–39]. Large‐scale genetic studies have also implicated *EEF1A2* as a candidate locus in epilepsy. Whole‐exome sequencing has found *EEF1A2* among the top genes associated with genetic generalized epilepsy, although not reaching genome‐wide significance [40]. Structural variations have also provided evidence of the involvement of *EEF1A2* in epilepsy, as copy number variants (CNVs) including *EEF1A2*, such as 20q13.33 microduplications, have been identified in epilepsy cohorts and validated using molecular methods [41]. Further cohort data have also provided evidence of the involvement of CNVs in epilepsy and their possible association with different phenotypes, including neonatal seizures with relatively better outcomes, indicating the possible modulatory role of *EEF1A2* in epilepsy phenotypes and outcomes [42]. Case reports have also indicated the variable nature of epileptic phenotypes associated with *EEF1A2* variants, ranging from drug‐resistant seizures and developmental delay to milder phenotypes [36, 43]. Notably, recurrent variants such as E122K have been associated with infantile‐onset myoclonic seizures, developmental stagnation, and characteristic electroencephalography (EEG) findings (e.g., continuous parietal delta activity), supporting a genotype‐linked but variably expressed clinical spectrum [36] Expanded cohorts further demonstrate that subsets of patients exhibit movement disorders and/or progressive neuroimaging abnormalities, suggesting broader network and, in some cases, neurodegenerative involvement within a neurodevelopmental spectrum [28, 35, 44].

Functional evidence for decreased de novo protein synthesis and altered regulation of the actin cytoskeleton provides a rationale for a mechanistic link between eEF1A2 dysfunction and synaptic stability, neuronal development, and neural‐network hyperexcitability leading to seizure and developmental delay phenotypes [14]. In aggregate, this evidence supports the role of *EEF1A2* as a critical gene in the molecular pathogenesis of DEE, with a need for further genotype–phenotype correlation and standardized phenotyping to advance current understanding of disease mechanisms and biomarkers.

Table 2 summarizes reported epilepsy‐related phenotype patterns associated with *EEF1A2* variants, integrating clinical features with their current evidence strength and interpretative context. It distinguishes recurring features from limited or emerging observations and points out gaps in need of further validation, including standardized phenotyping, electrophysiological verification, and prospective studies.

**Table 2:** eEF1A2‐related epilepsy phenotype patterns.

Pattern	Source	What is reported	Evidence strength	Interpretation	What is needed next
Early‐onset epilepsy with severe developmental impairment	[28]	Reported across multiple de novo EEF1A2 cohorts	Moderate to strong	Recurring DEE spectrum	Harmonized phenotyping; longitudinal outcomes
Continuous parietal delta activity on EEG	[36]	Reported in limited cases (notably with E122K)	Limited	Candidate EEG signature; not a validated biomarker	Replication; standardized EEG pipelines
Movement disorders in syndromic cases	[28]	Dystonia/choreoathetosis in expanded cohorts	Moderate	Network‐level dysfunction beyond seizures	Natural history studies; genotype‐stratified analysis
Progressive cerebral/cerebellar atrophy in some individuals	[28]	Degenerative course in subset	Moderate	Developmental and degenerative for a subset	Longitudinal imaging; mechanistic linkage

Abbreviations: DEE, developmental and epileptic encephalopathies; EEG, electroencephalography.

According to the evidence provided in Table 2, it cannot be assumed that eEF1A2 acts as a biomarker for the epilepsy‐related phenotypes. Some features, for example, early‐onset DEE patterns, show a moderate‐to‐strong association, whereas EEG patterns show limited evidence. Therefore, the eEF1A2‐related phenotypes support biological relevance and diagnostic consideration, but cannot be considered a biomarker due to a lack of validation, standardization, and replication.

### 5.3. Autism Spectrum Disorder (ASD), ID, and Related Phenotypes

De novo mutations in the *EEF1A2* gene are emerging as a cause of neurodevelopmental disorders, including ASD and ID. Whole‐exome sequencing of affected individuals with a consistent clinical presentation of severe to profound ID, autistic features, absent or limited speech, hypotonia in the neonatal period, epilepsy, and in some cases, progressive microcephaly, and dysmorphia, identified recurrent mutations such as p.Asp252His and p.Glu122Lys in affected individuals [28, 35, 39]. More case reports on individuals with movement disorders caused by particular mutations, for example, p.L246P, further expand the phenotypic spectrum of *EEF1A2* gene mutations [45]. Association studies on the normal population have also implicated *EEF1A2* in autism susceptibility. Particular single‐nucleotide polymorphisms (SNPs), for example, rs310619, have been associated with gene regulation, though not genome‐wide significant [46].

Functional evidence for disruption in protein synthesis and regulation in actin cytoskeletal pathways is provided as viable mechanistic substrates for these phenotypes [14]. Altered expression of *EEF1A2* has been observed in other neurodevelopmental disorders, including Fragile X syndrome, where significant upregulation is associated with dysregulated synaptic and translational pathways [47].

With respect to phenotype‐specific causality for *EEF1A2* variants, defined synaptic processes, and behavioral outcomes, these remain incompletely resolved; thus, mechanistic interpretations are provided as plausible and supported at the molecular and cellular levels.

### 5.4. Rett‐Like Presentations

De novo mutations in the *EEF1A2* gene have also been linked to Rett‐like phenotypes, which are indicative of the genetic heterogeneity of Rett syndrome, excluding canonical MECP2 mutations [48]. Investigations of patients with a progressive ID (Rett‐like manifestations) have revealed mutations in the *EEF1A2* gene and other neurodevelopmental genes, such as STXBP1 and ZNF238, including functional mutations in evolutionarily conserved regions such as the GTP‐binding region of the gene product [49, 50]. These Rett‐like manifestations should be included in the differential diagnosis of Rett syndrome when the major Rett genes are not mutated. However, due to a limited number of case reports, these manifestations should be considered part of the spectrum of neurodevelopmental disorders rather than an *EEF1A2*‐specific Rett syndrome.

The relationship between gain‐of‐function and loss‐of‐function mechanisms in *EEF1A2* variants can be reconciled. Dominant missense variants in *EEF1A2* can present an apparent paradox in which reduced canonical elongation activity (hypomorphic or loss‐of‐function effect) coexists with toxic cellular properties arising from dysregulated interactions or altered binding behavior. Functional analyses of common variants demonstrate decreased translation/elongation alongside altered actin bundling and neuronal morphological changes, supporting a model in which impaired canonical function and aberrant noncanonical coupling occur simultaneously [14]. Consistent with the variant–function relationships summarized in Table 1, these effects are not mutually exclusive, as reduced translation can coexist with cellular toxicity. At the cohort level, additional evidence suggests that haploinsufficiency and disruption of stress–response pathways may contribute to disease mechanisms in certain variant syndromes, although pathogenic effects are likely variant‐ and context‐dependent [28].

## 6. Neurodegenerative Disorders Where eEF1A2 Is Mechanistically Implicated

### 6.1. Evidence Summary

Unlike neurodevelopmental disorders, there is limited direct Mendelian evidence supporting the role of the *EEF1A2* gene in neurodegenerative disorders. However, the most common evidence is usually based on the pathways in cellular models such as the modulation of oxidative stress, autophagy, or mitophagy regulation, and injury response signaling [23, 29, 51]. Accordingly, these findings are framed as mechanistic hypotheses supported by model‐based evidence rather than as direct causal relationships.

### 6.2. Roles of eEF1A2 in PD

The *EEF1A2* gene is involved in the survival of dopaminergic neurons by regulating oxidative stress resistance and autophagy/mitophagy in models of PD. With regard to cell models, eEF1A2 is associated with the regulation of oxidative stress resistance by interacting with antioxidant systems [23]. eEF1A2 was shown to interact with PRDX1, an antioxidant enzyme that detoxifies reactive oxygen species, which in turn enhances resistance to peroxide‐induced oxidative stress, reduces caspase activation, and enhances prosurvival Akt signaling [23–27]. With regard to toxin‐induced models of PD, including 6‐OHDA and MPP^+^‐induced models, eEF1A2 expression is dynamically regulated in the context of neuronal stress, and the disruption of its association with antioxidant systems worsens oxidative damage and neuronal death [23, 26, 27].

In MPP^+^‐based dopaminergic cell models, knockdown of eEF1A2 results in reduced autophagy markers and makes the cells more susceptible to mitochondrial damage and *α*‐synuclein formation, indicating the protective role of eEF1A2 in autophagy/mitophagy in toxin stress conditions [29]. More precisely, knockdown of *EEF1A2* in differentiated SH‐SY5Y cells results in reduced LC3 puncta, LC3‐II/LC3‐I ratio, and phosphorylated Beclin‐1 levels, indicating impaired autophagic flux and makes the cells more susceptible to mitochondrial damage, defective mitophagy, and *α*‐synuclein formation [29]. This is also associated with decreased Akt1 and mTORC1 signaling, as well as increased levels of proapoptotic markers such as p53 and caspase‐3. This demonstrates that there is a protective role of eEF1A2 in maintaining homeostasis in neuronal cells that are exposed to stress [30]. Moreover, in models of PD that involve LRRK2, there have also been suggestions from the transcriptome analysis that eEF1A2 is significantly modulated in pathways that have been associated with neuronal survival, which suggests that eEF1A2 could counteract neurodegenerative signals in models of PD [52, 53].

Thus, the present studies indicate the protective role of eEF1A2 in PD models of oxidative stress resilience and autophagy/mitophagy–mediated neuronal survival; however, these findings are derived primarily from experimental systems and do not indicate eEF1A2 as a primary genetic cause of PD.

### 6.3. Roles of eEF1A2 in Motor Neuron Degeneration

The *wasted* (*wst/wst*) mouse is a key model that shows the necessity of eEF1A2 in motor neuron maintenance and neuromuscular junction function [9]. Homozygous mice exhibit tremors and gait abnormalities shortly after weaning, which progress quickly to motor neuron degeneration, paralysis, and early mortality [9]. Histopathological studies have demonstrated that there is a progressive sequence of neuromuscular degeneration that begins with early denervation of motor endplates, motor nerve terminal retraction, followed by gliosis, motor neuron vacuolation, and neurofilament accumulation in the spinal cord [54]. This study shows that loss of eEF1A2 function following postnatal reliance transition leads to progressive neuromuscular degeneration.

Mechanistically, the degeneration observed in the *wst/wst* mice follows a “dying‐back” neuropathy, where the loss of synapses in the neuromuscular junction leads to axonal and neuronal degeneration, and this type of degeneration is distinct from Wallerian degeneration [10]. This degeneration is also characterized by changes in pathways involved in neuronal survival, including a reduction in the neuropathy‐associated protein ZPR1, and this effect is also supported by evidence of delayed Wallerian degeneration [10].

Consequently, these findings support the role of eEF1A2 in the integrity of motor neurons and, as such, the contribution of this gene to neurodegenerative diseases, although caution should be taken in the interpretation of this effect in relation to human diseases.

### 6.4. Roles of eEF1A2 in Huntington′s Disease (HD)

HD is a neurodegenerative disease that is associated with CAG repeat expansions in the huntingtin gene, leading to altered cell functions as a result of the expression of the mutant huntingtin protein [55]. A study carried out in the brain cortex of mice showed that the mutant huntingtin protein specifically interacted with proteins that are involved in translation, such as eEF1A2, MAPK3, and EIF3H, suggesting that eEF1A2 may play a role in the translational dysregulation observed in HD pathology [56]. The disruption in its interaction is likely to be responsible for the impaired translation and neuronal degeneration associated with HD pathology.

Although it is evident that eEF1A2 is associated with the translational dysregulation associated with HD and that it interacts with mutant huntingtin protein, it is important to note that these findings are derived from experimental models and need further validation in human subjects.

### 6.5. Roles of eEF1A2 in AD

The involvement of eEF1A2 in the pathways of AD has been reported in the context of protein synthesis and the regulation of oxidative stress. In experimental studies of APPswe‐expressing N2a cells, the interaction of eEF1A2 with the Nrf2 pathway was reported [57]. The Nrf2 pathway is one of the most important pathways in antioxidant defense, and the modulation of the eEF1A2‐Nrf2 interaction affects antioxidant gene expression and reduces oxidative damage.

Additional evidence supports that the upregulation of the ubiquitin‐editing enzyme A20, which is regulated by Nrf2, can lead to decreased levels of eEF1A2 through increased ubiquitination, which in turn reduces cell death in models of oxidative injury [51]. These data support the role of eEF1A2 in a regulated process that balances the response to oxidative stress with protein homeostasis, with deregulation of this process potentially leading to neuronal dysfunction in AD.

Thus, eEF1A2 could be considered to be part of the modulatory system that integrates the pathways of translational control and oxidative stress in the context of AD pathogenesis, although its exact contribution to the disease process is not clearly defined. Since the evidence is largely derived from in vitro and animal models, the contribution of eEF1A2 to the pathophysiology of AD in humans should be considered with caution.

### 6.6. Roles of eEF1A2 in Ischemia‐Reperfusion Injury

Preclinical studies have suggested that eEF1A2 is involved in the process of cerebral ischemia/reperfusion injury (CIRI) via the Nrf2/A20/eEF1A2 signaling pathway. This signaling pathway modulates the process of oxidative stress, pyroptosis, and inflammatory reactions [51]. In the in vivo and in vitro experimental models of CIRI, the neuroprotective effects of biliverdin were mediated by the activation of Nrf2. The activation of Nrf2 upregulates the activity of the ubiquitin‐editing enzyme A20 and promotes ubiquitination‐mediated downregulation of eEF1A2, thereby reducing infarct size and suppressing pyroptosis [51]. The functional perturbation studies also indicated that the knockdown of A20 and the overexpression of EEF1A2 attenuate these protective effects, supporting a key role of eEF1A2 in the modulation of this pathway [51].

Similarly, ginsenoside Rb1 has been reported to exert neuroprotective activities in CIRI models via the same pathway, resulting in reduced neuronal injury, reduced inflammatory mediators, and inhibition of the NLRP3 inflammasome [58]. However, this was prevented by inhibition of Nrf2 or overexpression of eEF1A2. Overall, the data place eEF1A2 as a modulator of antioxidant and proinflammatory pathways in models of ischemic injury.

These data should be construed as mechanistic model evidence, considering the current reliance on experimental models. The clinical significance of eEF1A2 in human ischemic stroke and inflammatory brain injury needs to be determined, including its potential as a therapeutic target.

## 7. Conclusion

eEF1A2 is best conceptualized as a translation–cytoskeleton–stress integrator in neurons. For neurodevelopmental disease, replicated de novo pathogenic *EEF1A2* variants and functional validation support a causal role for eEF1A2 in DEE and related neurodevelopmental phenotypes. In neurodegenerative paradigms, evidence remains primarily pathway‐level and model‐derived, implicating eEF1A2 in oxidative stress resilience, autophagy or mitophagy regulation, and injury–response signaling. Priority gaps include defining neuron type–specific translation processes that are most sensitive to eEF1A2 perturbation using approaches such as ribosome profiling and cell type–specific translatome capture. In addition, further work is needed to clarify how PTM states relate to in vivo disease phenotypes beyond synaptic plasticity contexts. Finally, candidate clinical biomarkers require validation in larger, genotype‐stratified cohorts.

## Author Contributions

K.K. and A.P.: literature review, manuscript drafting, and editing. P.D.: conceptualization, literature review, manuscript drafting and editing, visualization, and supervision.

## Funding

No funding was received for this manuscript.

## Disclosure

All authors read and approved the final manuscript. The authors take full responsibility for the content of the publication.

## Conflicts of Interest

The authors declare no conflicts of interest.

## Data Availability

Data sharing is not applicable to this article as no new data were created or analyzed in this study.
